# Retraction Note: New Acetamidine Cu(II) Schiff base complex supported on magnetic nanoparticles pectin for the synthesis of triazoles using click chemistry

**DOI:** 10.1038/s41598-025-33778-x

**Published:** 2025-12-30

**Authors:** Hossein Khashei Siuki, Pouya Ghamari Kargar, Ghodsieh Bagherzade

**Affiliations:** Department of Chemistry, Faculty of Sciences, University of Birjand, Birjand, 97175‑615 Iran

Retraction of: *Scientific Reports* 10.1038/s41598-022-07674-7, published online 08 March 2022

The Editors have retracted this Article. After publication, significant concerns were raised regarding several figures presented in this work. Specifically, patterns of repeated signals and data duplication were identified in Figure 7. The Authors were unable to provide a satisfactory explanation or supporting data to address these concerns.

Therefore, the Editors no longer have confidence that the conclusions presented are adequately supported by the data.

Ghodsieh Bagherzade agrees with this retraction, Hossein Khashei Siuki does not agree with this retraction, and Pouya Ghamari Kargar did not explicitly state whether he agrees or disagrees with this retraction.

